# Diagnostic Accuracy and Optimal Use of Three Tests for Tuberculosis in Live Badgers

**DOI:** 10.1371/journal.pone.0011196

**Published:** 2010-06-17

**Authors:** Julian A. Drewe, Alexandra J. Tomlinson, Neil J. Walker, Richard J. Delahay

**Affiliations:** 1 Centre for Emerging, Endemic and Exotic Diseases, Royal Veterinary College, London, United Kingdom; 2 Food and Environment Research Agency, Woodchester Park, Gloucestershire, United Kingdom; Statens Serum Institute, Denmark

## Abstract

**Background:**

Accurate diagnosis of tuberculosis (TB) due to infection with *Mycobacterium bovis* is notoriously difficult in live animals, yet important if we are to understand the epidemiology of TB and devise effective strategies to limit its spread. Currently available tests for diagnosing TB in live Eurasian badgers (*Meles meles*) remain unvalidated against a reliable gold standard. The aim of the present study was to evaluate the diagnostic accuracy and optimal use of three tests for TB in badgers in the absence of a gold standard.

**Methodology/Principal Findings:**

A Bayesian approach was used to evaluate the diagnostic accuracy and optimal use of mycobacterial culture, gamma-interferon assay and a commercially available serological test using multiple samples collected from 305 live wild badgers. Although no single test was judged to be sufficiently sensitive and specific to be used as a sole diagnostic method, selective combined use of the three tests allowed guidelines to be formulated that allow a diagnosis to be made for individual animals with an estimated overall accuracy of 93% (range: 75% to 97%). Employing this approach in the study population of badgers resulted in approximately 13 out of 14 animals having their true infection status correctly classified from samples collected on a single capture.

**Conclusions/Significance:**

This method of interpretation represents a marked improvement on the current procedure for diagnosing *M. bovis* infection in live badgers. The results should be of use to inform future test and intervention strategies with the aim of reducing the incidence of TB in free-living wild badger populations.

## Introduction

The incidence of tuberculosis (TB) in cattle owing to infection with *Mycobacterium bovis* remains a cause for concern in large parts of Great Britain [1]. This places a considerable financial burden on the farming community and the government, and poses a potential zoonotic risk. Eurasian badgers (*Meles meles)* may maintain the disease and are a potential source of infection to cattle [2], [3]. Accurate diagnosis of *M. bovis* infection in badgers is critical if we are to understand the epidemiology of TB in this species and devise effective strategies to limit its spread to cattle.

Accurate diagnosis of TB in live animals is difficult. Pathogenesis varies between species and with the route of infection, resulting in a wide range of possible excretion pathways [4]. Intermittent excretion of *M. bovis* appears to occur in many species [5], [6], [7] hence the culture of clinical samples alone can be an insensitive indicator of an animal's infectiousness. Serologically-based assays may be hampered by delayed seroconversion [8], [9] and cross-reactivity with environmental mycobacteria [10]. In comparison, assays based on the measurement of cellular responses appear to produce better results [11]. One such test based on the detection of gamma interferon (IFNγ) from stimulated lymphocytes [12] has the advantage of being able to detect relatively early stages of infection with *M. bovis* [13]. However, none of the currently-available tests for *M. bovis* in live badgers has been validated against a reliable gold standard.

Until now, tests for diagnosing TB have usually been validated by trialling them on a population of animals ‘known’ to be either infected or uninfected (e.g., [14], [15], [16]). There are obvious problems with this method if the test used to define the infection status of the reference animals is itself imperfect. Most (if not all) tests for diagnosing TB in badgers have been validated against a standard postmortem procedure (e.g., [12], [16], [17]). The sensitivity of a standard postmortem procedure (including mycobacterial culture) for detecting *M. bovis* relative to a more detailed protocol was estimated to be around 54% in a study of 205 badgers [18]. The actual sensitivity is likely to have been even lower given that the detailed postmortem protocol could have itself missed several cases of infection. Two recent studies on separate badger populations in the UK [19] and Ireland [20] both found more than 60% of badgers infected with *M. bovis* did not have visible lesions. Postmortem examination (even with culture of tissue samples) is therefore an imperfect method for detecting infection and the use of it as a gold standard against which to judge the performance of other diagnostic tests may introduce significant error.

One solution where no ideal reference method of diagnosis exists is latent class analysis [21], [22]. This statistical approach, based on Bayes' theorem of conditional probability [23], still assumes that animals can be characterised by a dichotomous infection status (i.e. infected or not infected) but this status does not need to be known. This allows diagnostic tests to be validated without the assumption of a gold standard [24], [25]. *A priori* belief concerning the true values of parameters, incorporating uncertainty, is quantified (prior distributions) based on previous knowledge of test performance and updated by the addition of empirical data (expressed in the likelihood) to generate modified estimates (posterior distributions) of said parameters. For estimations of diagnostic test accuracy, these parameters may be test sensitivity (the proportion of infected animals correctly identified by the test), test specificity (the proportion of non-infected animals correctly identified by the test) and infection prevalence (the proportion of the population that is infected). If no prior knowledge of true infection status or prevalence is available, uniform prior distributions (“flat” probability distributions with equal probability assigned to a large range of parameter values) may be used [26]. By including information on multiple tests simultaneously, estimates for the performance of each test are modified in light of the others. Important assumptions of this approach are that test sensitivity and specificity are the same in all populations, and tests are conditionally independent of each other. Conditional independence implies that for any given animal that is infected (or not), the probability of a positive (or negative) outcome for test A is the same regardless of a known outcome for test B [24]. If this latter assumption is not true, a co-dependence term should be included to avoid bias [24], [27]. This Bayesian method allows the performance of diagnostic tests to be estimated in the absence of a gold standard, in situations where analysis by traditional methods would have led to considerable error [28].

The present study had two aims. The first was to evaluate the performance of three diagnostic tests for TB in live badgers, using a Bayesian approach in the absence of a reference test. The second aim was to use these estimates to determine guidelines for the optimal implementation of these tests, either singly or in combination, to maximise the accuracy of diagnosis of TB in live badgers. These guidelines may have useful applications to field research projects and the development of intervention strategies involving the use of live tests to manage TB in badger populations.

## Methods

### Ethics statement

Trapping, anaesthesia and biological sampling of badgers were carried out under licence from the UK Home Office (licence number PPL60/3609) according to the Animals (Scientific Procedures) Act 1986. All procedures were approved by the Food and Environment Agency Ethical Review Panel.

### Study site and sample collection

Data and samples were collected from wild badgers living in the Woodchester Park study area, a 7 km^2^ region of Cotswold limestone escarpment in Gloucestershire, south-west England (51°43′N, 2°16′W). The resident population of badgers (approximately 300 individuals in 26 social groups) has been the subject of long-term research into badger ecology and TB epidemiology, details of which are given elsewhere [29]. Badgers were captured in the immediate vicinity of their setts in peanut-baited cage traps and transported to a sampling facility to be anaesthetised and examined. All animals were anaesthetised by intramuscular injection of a combination of 8 mg/kg ketamine hydrochloride (Vetalar; Pfizer Ltd, Sandwich, UK), 0.04 mg/kg medetomidine hydrochloride (Domitor; Pfizer Ltd) and 0.8 mg/kg butorphanol tartrate (Torbugesic; Fort Dodge Animal Health, Southampton, UK) [30]. They were then sexed, weighed and measured. On first capture each badger was given a unique identifying tattoo on its ventral abdomen [31] which allowed individuals to be identified thereafter. Samples of faeces, urine, tracheal aspirate, oesophageal aspirate and swabs from bite wounds (where present) were collected for mycobacterial culture and up to 12 ml of jugular blood was taken for serological and gamma interferon testing (see below). After recovery from anaesthesia, badgers were released at the site where they had been captured. Each social group was trapped four times per year. The present study used data derived from 875 capture events that occurred between July 2006 and December 2008, which represented 305 individual badgers (130 male, 175 female) from 26 social groups. Of the badgers caught, individuals were sampled on average three times (range 1 to 10) during the study period. All diagnostic tests gave conclusive results on each of the 875 sampling sessions included in the dataset.

### Mycobacterial culture

All samples (except blood) were individually cultured for mycobacteria using standard techniques [32]. Briefly, samples were decontaminated with 10% oxalic acid, centrifuged, and the pellet inoculated in triplicate onto modified Middlebrook 7H11 agar slopes. Cultures were incubated at 37°C±2°C for at least 6 weeks. Any growth of organisms characteristic of mycobacteria was identified as *M. bovis* by spoligotyping [33]. Positive and negative controls were always included. One or more positive culture results were interpreted as indicative of current infection with *M. bovis*.

### Gamma interferon assay

Whole heparinised blood was subjected to an IFNγ assay as reported previously [12]. This test of cell-mediated immunity is based on the stimulation of lymphocytes in whole-blood culture and the subsequent detection of IFNγ by sandwich ELISA [12]. A positive result was taken to indicate previous or current infection with *M. bovis.*

### Serological assay

A commercially available lateral flow immunoassay (BrockTB Stat-Pak; Chembio Diagnostic Systems, New York, USA) was used to examine badger serum for IgM and IgG antibodies to *M. tuberculosis*–complex antigens MPB83, ESAT-6 and CFP10 [15]. Antigen-conjugated blue latex particles bound with antibody (if present in the serum sample) to form a coloured immune complex that was visible as a blue band in the test window. A control band in the test window indicated the assay had functioned correctly. A positive serological result was interpreted as evidence of previous or current infection with *M. bovis.*

### Statistical analysis

The sensitivity and specificity of each of the three diagnostic tests (mycobacterial culture, IFNγ and Stat-Pak) and the prevalence of *M. bovis* infection in the study population were estimated in the absence of a gold standard using Bayesian methods [25], [34]. An assumption of conditional independence of all tests was made due to their differing biological mechanisms of action (culture examines for presence of the pathogen, IFNγ measures cell-mediated immunity, and Stat-Pak examines for presence of antibody) and so no co-variance parameters were included in the model [27]. Prior information about test sensitivities and specificities and prevalence of infection was quantified using beta (α, β) distributions. Beta distributions are bounded by 0 and 1 and are thus suited to modelling binomial probabilities in a Bayesian analysis [34]. BetaBuster software (downloadable from http://www.epi.ucdavis.edu/diagnostictests/betabuster.html) was used to calculate beta distributions from published estimates of the sensitivity and specificity of IFNγ [12] and Stat-Pak [16] (when assessed against the current gold standard of *M. bovis* culture from postmortem tissue samples) and expert-elicited estimates of the most likely (modal) values for culture (Table 1). The likely prevalence of *M. bovis* infection in the study population was estimated from historical data to be approximately 24%, with a 2.5–97.5 percentile range of 16–35%. This information equated to a prior beta (19.26, 58.83) distribution for prevalence. Prevalence was not in itself the focus of this study and was included in the model solely to facilitate estimation of sensitivity and specificity of the three diagnostic tests.

**Table 1:** Values of priors and corresponding beta distributions used to estimate the performance of three diagnostic tests for *M. bovis* infection in live badgers.

Diagnostic test	Parameter	Mode	2.5^th^–97.5^th^ percentile range	Beta (α, β) prior distribution	Source of prior probabilities
Culture	Se	0.100	0.025, 0.373	2.25, 12.26	A. Tomlinson (unpubl. data)
	Sp	0.999	0.939, 0.999	60.61, 1.06	M. Chambers (pers. comm.)
IFNγ	Se	0.809	0.640, 0.901	26.41, 7.00	Ref [12]
	Sp	0.936	0.621, 0.987	9.95, 1.61	Ref [12]
Stat-Pak	Se	0.492	0.431, 0.553	127.02, 131.12	Ref [16]
	Sp	0.931	0.622, 0.986	10.22, 1.68	Ref [16]

Se = sensitivity.

Sp = specificity.

The freeware program WinBUGS 1.4.3 [35] was used to run all models using Gibbs sampling. A Markov chain Monte Carlo simulation was conducted to estimate the median and 95% probability intervals (also known as credibility intervals) for all parameters of interest from the respective posterior distributions. Estimates were generated from 50,000 iterations after discarding an initial burn-in of 5,000 iterations. Convergence for each model was assessed by simultaneously running five chains from different starting values and visually checking time-series plots of selected variables as well as Gelman-Rubin diagnostic plots [36] for each parameter.

A sensitivity analysis using vaguer (partially informative) priors was performed to test the repeatability of results as well as the degree of reliance on the prior distributions. Prior beta distributions were changed to uniform (a, b) distributions, where: a = 0, b = 0.5 for culture sensitivity; a = 0.25, b = 0.75 for Stat-Pak sensitivity; and a = 0.5, b = 1 for IFNγ sensitivity and the specificities of all three tests. All median estimates of test sensitivity and specificity fell within 4% of the original values, except for Stat-Pak sensitivity, which increased by 18%.

For each of the three tests, positive and negative predictive values, likelihood ratios and post-test probabilities of infection were calculated [37] given the estimated prevalence of infection in the badger population. Tests were interpreted individually and in parallel (whereby two or more different tests were run concurrently and a positive diagnosis was made if at least one test gave a positive result). A glossary of terms relating to diagnostic test performance, together with their derivations, is given in Table S1.

## Results

### Test results

**Figure 1:**
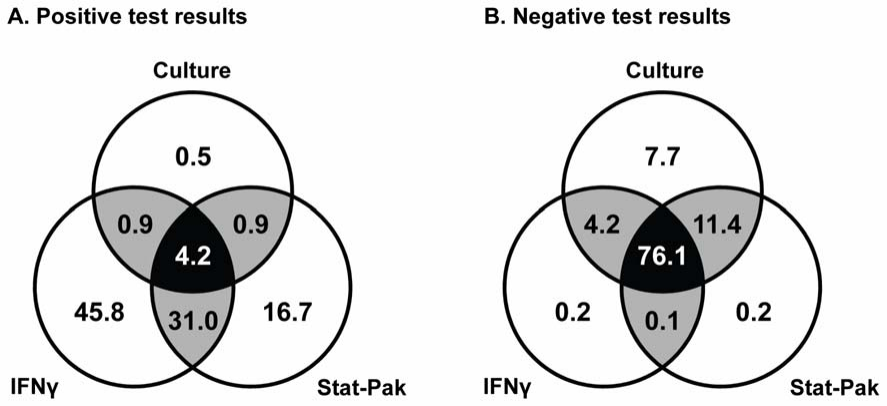
The degree of agreement between three tests for *M. bovis* infection in live badgers. (A) Percentage agreement between positive test results (n = 216 occurrences where at least one test gave a positive result); (B) percentage agreement between negative test results (n = 866 occurrences where at least one test gave a negative result).

The cross-classified results of the three diagnostic tests are presented in Table 2. Fourteen of 875 samples (1.6%) cultured positive for *M. bovis*, whereas 177 (20.2%) tested positive using IFNγ, and 114 (13.0%) gave a positive Stat-Pak result. The degree of inter-test agreement for positive and negative test results is shown in Figure 1. The data show that it was rare for all three tests to agree on a positive result: in only 4.2% of cases of at least one positive result were all three tests positive (Figure 1a). In 31% of cases both IFNγ and Stat-Pak were positive but culture was negative, and 45.8% of the time the IFNγ result was positive when the other two tests were negative (Figure 1a). A different trend was seen in agreement between negative test results with the highest level of agreement (76.1%) occurring when all three tests gave the same (negative) result (Figure 1b). Negative results rarely occurred in just one test: for example, IFNγ was negative when both culture and Stat-Pak were positive in only 0.2% of cases (Figure 1b).

**Table 2:** Cross-classified observed results of three diagnostic tests for *M. bovis* infection performed on 875 sets of samples collected from 305 live badgers.

	Culture+		Culture−	
	Stat-Pak+	Stat-Pak−	Stat-Pak+	Stat-Pak−
**IFNγ+**	9	2	67	99
**IFNγ−**	2	1	36	659

+ = positive test result.

− = negative test result.

### Sensitivity and specificity

**Figure 2:**
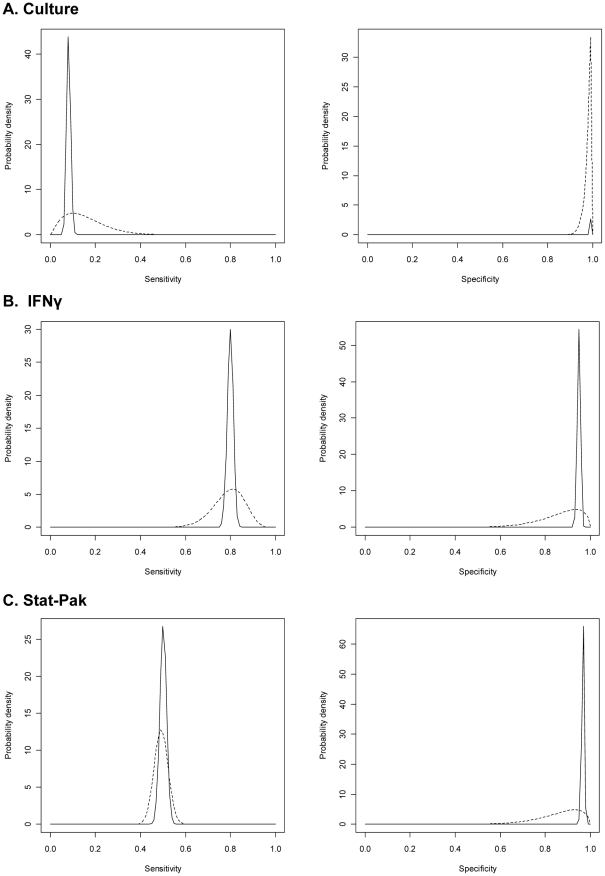
Beta distributions for sensitivity and specificity estimates of three tests for *M. bovis* in badgers. Dashed lines indicate prior distributions. Solid lines indicate posterior distributions.

Estimates for the sensitivity and specificity of the three tests, determined by Markov chain Monte Carlo simulation, appear in Table 3. The posterior medians represent the estimates of diagnostic test performance when each test was used independently in the absence of a known reference test. Culture was of low sensitivity (8.0%) but very high specificity (99.8%). IFNγ showed better sensitivity (79.9%) and good specificity (95.0%). Stat-Pak had a sensitivity of 50.4% and a specificity of 96.9%. The prior and posterior distributions for each test parameter are plotted in Figure 2. The prior distributions were updated by iterations of the empirical test result data to produce the narrower posterior distributions.

**Table 3:** Prior and posterior median and 95% probability intervals (PI) for estimates of sensitivity and specificity of three diagnostic tests for *M. bovis* infection in live badgers.

	Sensitivity				Specificity			
	Prior median	Prior 95% PI	Posterior median	Posterior 95% PI	Prior median	Prior 95% PI	Posterior median	Posterior 95% PI
**Culture**	0.139	0.025, 0.373	0.080	0.045, 0.130	0.988	0.939, 0.999	0.998	0.993, 1.000
**IFNγ**	0.796	0.640, 0.909	0.799	0.688, 0.895	0.881	0.621, 0.987	0.950	0.914, 0.985
**Stat-Pak**	0.492	0.431, 0.553	0.504	0.449, 0.561	0.879	0.622, 0.986	0.969	0.946, 0.991

### Diagnostic accuracy of tests

The performance of each of the tests when used singly and in combination is presented in Table 4. The test with the highest positive predictive value was culture (93%) and this was not improved by interpreting it in parallel with any other tests. IFNγ gave the best negative predictive value of any single test (95%) and this was further slightly improved by interpreting this test in parallel with Stat-Pak (combined negative predictive value = 97%). Addition of mycobacterial culture so that all three tests were interpreted in parallel did not improve the negative predictive value (Table 4).

**Table 4:** Performance of the three diagnostic tests when used singly and in combination to detect *M. bovis* infection in live badgers.

Diagnostic test(s)	Positive predictive value	Negative predictive value	Likelihood ratio of a positive test	Likelihood ratio of a negative test	Post-test probability of infection given a positive test result	Post-test probability of infection given a negative test result
Culture	0.93	0.81	50	0.92	0.93	0.20
IFNγ	0.81	0.95	16	0.21	0.81	0.05
Stat-Pak	0.81	0.88	16	0.51	0.81	0.12
Culture & IFNγ *	0.81	0.95	16	0.19	0.81	0.05
Culture & Stat-Pak *	0.82	0.89	17	0.47	0.82	0.11
Stat-Pak & IFNγ *	0.75	0.97	11	0.11	0.75	0.03
Culture & IFNγ & Stat-Pak *	0.75	0.97	11	0.10	0.75	0.03

* =  Parallel interpretation of tests.

Whilst likelihood ratios remain unaffected by infection prevalence, the same is not true for predictive values and hence estimates of the latter are specifically related to the observed prevalence of *M. bovis* in the study population. Prevalence of *M. bovis* infection was estimated from the posterior distribution at 20.8% (95% probability interval: 16.4–25.8%). Given this estimate, the highest post-test probability of infection given a positive test result was for culture, at 93% (Table 4). The lowest post-test probability of infection given a negative test result was for the IFNγ and Stat-Pak combination (3%), meaning that obtaining a negative result in both these tests represents a 97% likelihood of freedom from infection.

**Figure 3:**
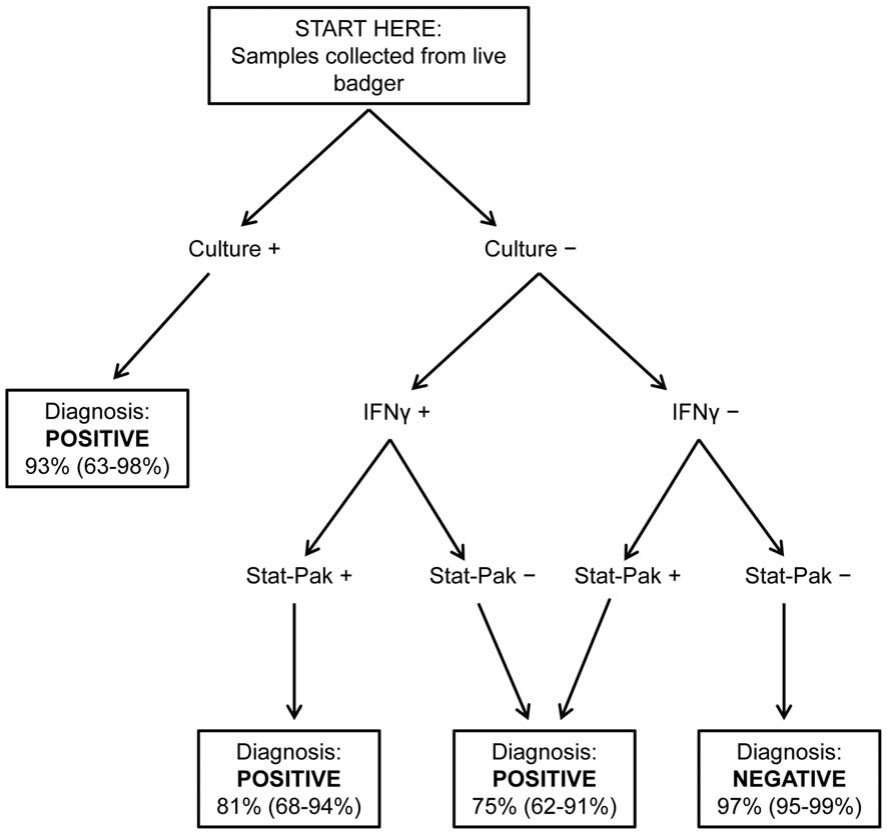
Decision tree illustrating the optimal use of three tests for detecting *M. bovis* infection in live badgers. The three tests are run concurrently and the culture result is interpreted first, followed by IFNγ and Stat-Pak. Percentage figures in boxes are median (and 95% probability interval) estimates of the level of confidence associated with each diagnosis.

Using these calculations, we formulated guidelines for the optimal use and interpretation of the three tests (Figure 3). For each badger, all three tests should be run concurrently and the results of IFNγ and Stat-Pak interpreted in parallel. The high positive predictive value of culture means that any badger generating a positive culture result can be considered infected with *M. bovis* with an estimated probability of 93%. For culture-negative badgers, the post-test probability of infection ranges from 81% (when both IFNγ and Stat-Pak are positive) to 75% (if either IFNγ or Stat-Pak is positive) to 3% (when both IFNγ and Stat-Pak are negative) (Figure 3). The overall level of diagnostic error is just 7.4% (95% probability interval: 2.6–12.5%) when this method of interpretation is used, given the observed proportions of badgers with each combination of test results (Table 2). Thus, by employing this approach at the estimated prevalence of infection, approximately 13 out of 14 badgers in this population will have their true infection status correctly classified from samples collected on a single capture.

**Figure 4:**
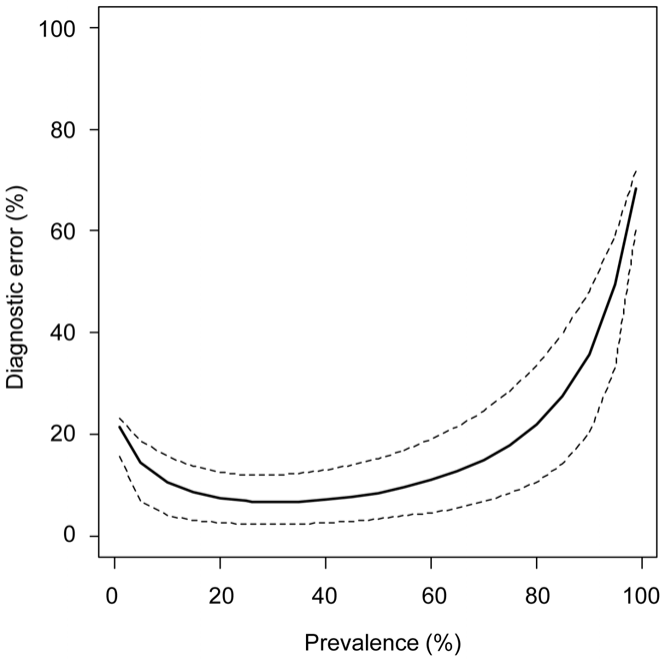
Effect of variation in *M. bovis* prevalence on the accuracy of diagnosis of infection in live badgers. The estimated overall diagnostic error (solid line) together with 95% probability intervals (dashed lines) are shown, based on application of the protocol illustrated in Figure 3.

To quantify the effect of variation in infection prevalence on diagnostic error, a range of theoretical prevalence values were examined (Figure 4). This analysis indicated that a reduction in infection prevalence from 20.8% to 10% would be accompanied by an increase in overall diagnostic error from 7.4% to 10.6%. Conversely, an increase in infection prevalence from 20.8% to 30% would be associated with a reduction in overall classification error from 7.4% to 6.7%. Diagnostic accuracy was highest when the prevalence of *M. bovis* in the study population was 30% (Figure 4).

## Discussion

The purpose of this study was to determine the accuracy and optimal use of three diagnostic tests for TB in live badgers. Bayesian methods were used to estimate test performances in the absence of a gold standard and without knowledge of the true infection status of individual badgers. Although no single test was judged to be sufficiently sensitive and specific to be used in isolation, selective combined use of the three tests allowed a diagnosis to be made with an estimated overall confidence level of 93% (95% probability interval: 87–97%) and this was not affected by moderate variations in prevalence. This method of interpretation represents a marked improvement on procedures based on single tests for diagnosing *M. bovis* infection in live badgers.

The results of the sensitivity analysis for all but one measure of test performance (Stat-Pak sensitivity) fell within 4% of the original model results, despite having substituted partially informative uniform distributions for the prior estimates obtained from the literature [12], [16] and expert opinion. This suggests that the chosen prior distributions were appropriate. Indeed, the posterior estimates of sensitivity and specificity for both Stat-Pak and IFNγ closely matched the published priors (Tables 1 and 3). However, when a uniform prior distribution was used to estimate Stat-Pak sensitivity, the median of the posterior distribution for this parameter increased by 18%. This indicates that the posterior estimate for Stat-Pak sensitivity of 50.4% was not solely derived from the empirical data, and the prior distribution used in the model strongly influenced the posterior estimate of this parameter. The model that was used to estimate the seven parameters (sensitivity and specificity for each of the three tests plus infection prevalence) contained seven degrees of freedom (independent data cells in Table 2) and so should have been ‘identifiable’, that is, it should have converged to the ‘true’ values [38]. One possible explanation comes from the observed differences in positive results between the three tests (Figure 1a), and in particular the large number of cases where badgers tested positive on IFNγ but negative on Stat-Pak (Table 2). The higher number of badgers testing IFNγ positive meant that the posterior estimate of IFNγ specificity increased more than the posterior estimate of Stat-Pak sensitivity (Table 3). A further possibility is that the prior modal estimate of Stat-Pak sensitivity was overly cautious, and therefore the actual estimate may be somewhat higher than reported here. Re-running the model in the future when more Stat-Pak negative results are available should resolve this issue. Nonetheless, the results of the present analysis appear to be epidemiologically plausible and this paper has established the concept of applying Bayesian analysis to determine the accuracy of three tests for TB in live badgers. To the best of our knowledge, this is the first published application using a Bayesian approach to the diagnosis of TB in badgers.

This study focused on estimating diagnostic test performance and not on infection prevalence. Had the aim of this study been to estimate infection prevalence, it would have been necessary to account for bias arising from any effect of a badger's infection status on the probability of that animal entering a trap and being sampled. A chi-squared analysis of individual badger trapping frequency revealed no significant relationship between TB status and likelihood of subsequent capture (χ^2^ = 11.36, df = 6, p = 0.08). Thus, any bias that may have been introduced to the prevalence estimate by including multiple testing of individuals is unlikely to have affected the parameters of interest. The fact that this p-value approaches significance could reflect an increased infection risk with age. Alternatively, the sensitivity of TB detection in an infected badger might be increased by multiple captures (and testing) of that animal. A further possibility is that infected badgers may be more likely to enter traps.

The accuracy of diagnostic tests for TB in live animals is likely to vary with the stage of disease. Several studies have attempted to quantify the influence of disease severity on the accuracy of diagnostic tests [14], [16], [39], [40], [41], [42]. A positive correlation was found between the sensitivity of the Stat-Pak serological assay and the time elapsed since experimental inoculation with *M. bovis* in a study of 25 cattle, with sensitivity increasing from 60% at 7 weeks to 96% at 18 weeks post-challenge [41]. In a study of naturally-infected badgers, sensitivity of the Stat-Pak assay was higher in individuals with disseminated TB than those with no visible lesions at subsequent postmortem examination [16]. The authors inferred that this indicated the test was useful for detecting badgers at greatest risk of transmitting disease. However, the authors acknowledged that animals with the most severe disease may not necessarily be those at greatest risk of transmitting infection [16]. Rather, risk is an interplay of several factors including the routes and levels of infection and excretion, the infectious dose, and the chance of encountering infection [4]. Therefore, whilst serologic tests may detect animals in the late stages of *M. bovis* infection [42], they are currently of limited use when used in isolation in disease control programmes since they fail to detect a large proportion of an individual's infectious period [39].

In the present study, a strong positive association between the results of culture and Stat-Pak was evident, with co-occurrence of a positive result in both these tests happening approximately six times as often as would be expected by chance (log-linear analysis using data from Table 2; β = 3.6, approximate z statistic = 2.9, p = 0.004). This association between the results of culture and Stat-Pak does not invalidate the assumption of conditional independence because tests that are quite different in their mechanism of action may still identify the same subpopulation of animals as infected. For example, two tests based on completely different principles of disease detection that were both 100% sensitive and 100% specific would show a perfect positive association in their results despite being conditionally independent. In the present study, it appears that culture and Stat-Pak detect a similar (probably late) stage of infection, whilst IFNγ detects a different subpopulation of badgers at a probable earlier stage of infection [12]. These findings suggest that using the Stat-Pak test in parallel with the IFNγ assay is likely to lead to improved sensitivity of TB diagnosis in live badgers. Positive associations were also observed in the present study between IFNγ and Stat-Pak (where a positive co-occurrence occurred 3.3 times as often would be as expected by chance: β = 2.5, approximate z statistic  = 10.8, p<0.001), and between IFNγ and culture (3.9 times that expected by chance: β = 2.6, approximate z statistic  = 2.1, p = 0.04). Although these associations were both weaker than the association between culture and Stat-Pak, they indicate that there is no clear-cut difference between the subpopulations of infected badgers identified by each of the tests.

In conclusion, Bayesian analysis of the performance of three diagnostic tests (mycobacterial culture, IFNγ and Stat-Pak) showed that they all provided valuable information to allow reasonably confident classification of TB status in live badgers. Two tests (culture and Stat-Pak) were limited by their low sensitivity when used independently. By running all three tests concurrently and interpreting the results of IFNγ and Stat-Pak in parallel, a high degree of diagnostic accuracy was achievable. This approach may be of value in the interpretation of test result data from field studies, in simulation modelling of live test-based intervention strategies, and in informing management policies with the aim of reducing TB incidence in free-living wild badgers, and, ultimately, in cattle.

## Supporting Information

Table S1 Glossary and derivation of terms relating to diagnostic test performance.
(0.06 MB DOC)
