# Supplementary material for: Diagnostic Accuracy and Optimal Use of Three Tests for Tuberculosis in Live Badgers
Source: PLoS One. 2010 Jun 17;5(6):e11196. doi: 10.1371/journal.pone.0011196 (PMC2887451; doi:10.1371/journal.pone.0011196)
Supplement: Table S1 — Glossary and derivation of terms relating to diagnostic test performance. (0.06 MB DOC) [file pone.0011196.s001.doc]

**Table S1. Glossary and derivation of terms relating to diagnostic test performance.**

| **Term** | **Symbol** | **Definition** | **Derivation** |
| --- | --- | --- | --- |
| **Prevalence of infection** | π | The probability before any test is carried out that an individual is infected. Also known as the prior probability of infection, prevalence equates to the proportion of the population that is infected. Prevalence may vary over time and between populations. | π =no. animals infected / population size |
| **Pre-test odds of infection** | Odds_pre_ | The prior probability of infection expressed as odds. | Odds_pre_ = π / (1 – π) |
| **Sensitivity** | Se | The proportion of infected animals (true positives) that are correctly identified by the test. | Se = true positives / (true positives + false negatives) |
| **Specificity** | Sp | The proportion of non-infected animals (true negatives) that are correctly identified by the test. | Sp = true negatives / (true negatives + false positives) |
| **Positive predictive value** | PPV | The proportion of test-positive animals that are actually infected. It is a revised estimate of the probability of infection in an animal given a positive test result. The PPV of a test is not universal since it varies with disease prevalence. | PPV = Se.π / [Se.π + (1 – Sp)(1 – π)] |
| **Negative predictive value** | NPV | The proportion of test-negative animals that are not infected. It is a revised estimate of the probability of an animal not being infected given a negative test result. Like the PPV, the NPV of a test is not universal since it varies with disease prevalence. | NPV = Sp(1 – π) / [π(1 – Se) + Sp(1 – π)] |
| **Likelihood ratio of a positive test** | LRPT | The LRPT compares the probability of obtaining a positive test result in an infected individual to the probability of obtaining the same result if they were uninfected. The *larger* the value of LRPT, the more useful that test is for increasing certainty about a positive diagnosis. Likelihood ratios do not vary with disease prevalence and so are stable expressions of test performance. | LRPT = Se / (1 – Sp) |
| **Likelihood ratio of a negative test** | LRNT | The LRNT compares the probability of obtaining a negative test result in an infected individual to the probability of obtaining the same result if they were not infected. The *smaller* the value of LRPT, the more useful that test is for increasing certainty about a negative diagnosis. The reciprocal (1 / LRNT) is perhaps more intuitive as it indicates the likelihood of obtaining a negative test result in a non-infected animal as opposed to an infected animal, with *larger* values indicating increased usefulness of a test. | LRNT = (1 – Se) / Sp |
| **Post-test odds of infection** | Odds_post_ | The posterior probabilities of infection following a positive or negative test result. | Odds_post_ given a positive test result = Odds_pre_ x LRPT.  Odds_post_ given a negative test result = Odds_pre_ x LRNT |
| **Post-test probability of infection** | Prob_post_ | Post-test odds expressed as a probability. | Prob_post_ =1/[1+(1/ Odds_post_)] |
